# Increased triacylglycerol - Fatty acid substrate cycling in human skeletal muscle cells exposed to eicosapentaenoic acid

**DOI:** 10.1371/journal.pone.0208048

**Published:** 2018-11-29

**Authors:** Nils G. Løvsletten, Siril S. Bakke, Eili T. Kase, D. Margriet Ouwens, G. Hege Thoresen, Arild C. Rustan

**Affiliations:** 1 Department of Pharmaceutical Biosciences, School of Pharmacy, University of Oslo, Oslo, Norway; 2 Centre of Molecular Inflammation Research, and Department of Cancer Research and Molecular Medicine, Norwegian University of Science and Technology, Trondheim, Norway; 3 German Diabetes Center, Leibniz Center for Diabetes Research, Heinrich Heine University, Medical Faculty, Duesseldorf, Germany; 4 German Center for Diabetes Research (DZD), München-Neuherberg, Germany; 5 Department of Endocrinology, Ghent University Hospital, Ghent, Belgium; 6 Department of Pharmacology, Institute of Clinical Medicine, University of Oslo, Oslo, Norway; University of Illinois, UNITED STATES

## Abstract

It has previously been shown that pretreatment of differentiated human skeletal muscle cells (myotubes) with eicosapentaenoic acid (EPA) promoted increased uptake of fatty acids and increased triacylglycerol accumulation, compared to pretreatment with oleic acid (OA) and palmitic acid (PA). The aim of the present study was to examine whether EPA could affect substrate cycling in human skeletal muscle cells by altering lipolysis rate of intracellular TAG and re-esterification of fatty acids. Fatty acid metabolism was studied in human myotubes using a mixture of fatty acids, consisting of radiolabelled oleic acid as tracer (^14^C-OA) together with EPA or PA. Co-incubation of myotubes with EPA increased cell-accumulation and incomplete fatty acid oxidation of ^14^C-OA compared to co-incubation with PA. Lipid distribution showed higher incorporation of ^14^C-OA into all cellular lipids after co-incubation with EPA relative to PA, with most markedly increases (3 to 4-fold) for diacylglycerol and triacylglycerol. Further, the increases in cellular lipids after co-incubation with EPA were accompanied by higher lipolysis and fatty acid re-esterification rate. Correspondingly, basal respiration, proton leak and maximal respiration were significantly increased in cells exposed to EPA compared to PA. Microarray and Gene Ontology (GO) enrichment analysis showed that EPA, related to PA, significantly changed i.e. the GO terms “Neutral lipid metabolic process” and “Regulation of lipid storage”. Finally, an inhibitor of diacylglycerol acyltransferase 1 decreased the effect of EPA to promote fatty acid accumulation. In conclusion, incubation of human myotubes with EPA, compared to PA, increased processes of fatty acid turnover and oxidation suggesting that EPA may activate futile substrate cycling of fatty acids in human myotubes. Increased TAG—FA cycling may be involved in the potentially favourable effects of long-chain polyunsaturated n-3 fatty acids on skeletal muscle and whole-body energy metabolism.

## Introduction

Fatty acids (FA) have many diverse functions ranging from being structurally the main component in cellular membranes, to play a central role in metabolic processes and acting as signaling molecules [1]. Long-chain n-3 polyunsaturated fatty acids (n-3 FA), such as eicosapentaenoic acid (EPA, 20:5n-3) has been given much attention during recent decades for their potentially beneficial effects on human health and diseases [2]. Among other things, n-3 FA exerts favourable effects on energy metabolism, including improvements in lipid metabolism and increased thermogenesis, and prevention of obesity [3–6].

In the mammalian cell there are certain processes that are entirely uncoupled,”futile”, and therefore thermogenic or energy consuming. It is well known that brown adipose tissue is a highly thermogenic organ wherein uncoupling protein 1 play a central role [7]. In comparison, skeletal muscle mass in human is much greater and potential futile cycling or uncoupling mechanisms in muscle could therefore have a substantially effect on regulating energy homeostasis as well as muscle function [7]. The energy lost by triacylglycerol—fatty acid (TAG—FA) cycling is an example of a substrate cycle in which esterification of TAG is followed by hydrolysis, leading to heat expenditure [8]. For instance, it has been observed that TAG—FA cycling plays an important role in controlling lipid metabolism *in vivo* in humans during and after exercise [9]. This cycle is also present *in vitro* in cells such as isolated human white adipocytes [10, 11], 3T3-L1 mice adipocytes [12] and C2C12 myotubes [13]. Further, it has been observed that a combined intervention using n-3 FA (docosahexaenoic acid (DHA) and EPA) and mild calorie restriction exerted synergism in the prevention of obesity in mice fed a high-fat diet. This was associated with strong hypolipidemic and insulin-sensitizing effects involving a futile substrate cycle in white adipose tissue based on lipolysis of intracellular TAG and re-esterification of fatty acids, in association with induction of mitochondrial oxidative phosphorylation capacity, fatty acid β-oxidation and increased energy expenditure [6, 14]. Thus, there are some mechanistic animal studies that suggest futile TAG—FA cycling induced by feeding n-3 FA/EPA, and that this might also occur in human skeletal muscle. We have previously observed that pretreatment of human skeletal muscle cells (myotubes) with EPA promoted uptake of FA, increased TAG accumulation and fatty acid β-oxidation while also stimulating glucose uptake and oxidation without changing insulin action [15, 16]. EPA was also shown to increase FA accumulation compared to palmitic acid (PA) and oleic acid (OA), to positively influence energy metabolism and metabolic switching of myotubes [15–17]. In addition, we have observed in human myotubes that the differences between OA and PA on their cellular accumulation and lipolysis were eliminated when co-incubated with EPA [18]. However, in that study we did not focus on the possibility of EPA to increase cellular TAG—FA cycling.

In the present study our focus was therefore to simultaneously examine the effect of EPA compared to PA on real-time fatty acid accumulation, lipolysis and re-esterification and on fatty acid oxidation and mitochondrial function to further explore if an increased TAG—FA turnover, e.g. futile substrate cycling, may be present in skeletal muscle and possibly be involved in the favourable effects caused by EPA on cellular energy metabolism.

## Materials and methods

### Materials

Dulbecco´s modified Eagles medium (DMEM-Glutamax), DMEM without phenol red, minimum essential media (αMEM), heat-inactivated fetal calf serum (FCS) and horse serum, and penicillin-streptomycin-amphotericin B were purchased from Gibco Invitrogen (Gibco, Life Technologies, Paisley, UK). Ultroser G was purchased from PALL Life Science (Port Washington, NY, US), insulin (Actrapid) from NovoNordisk (Bagsvaerd, Denmark), BSA (bovine serum albumin) (essentially fatty acid-free), Dulbecco’s phosphate-buffered saline (DPBS with Mg^2+^ and Ca^2+^), eicosapentaenoic acid (EPA, 20:5n-3), palmitic acid (PA, 16:0), oleic acid (OA, 18:1n-9), linoleic acid (LA, 18:2n-6), extracellular matrix gel, HEPES, A922500 (DGAT1 inhibitor) were all obtained from Sigma (St. Louis, MO, US).

[1-^14^C]oleic acid (58.2 mCi/mmol) was from PerkinElmer NEN (Boston, MA, US). Corning CellBIND tissue culture 96-well plates were obtained from Corning Life-Sciences (Schiphol-Rijk, The Netherlands). Biocoat 25 cm^2^ cell flasks were from BD Biosciences (Franklin Lakes, NJ, US) and 12-well plates from Corning Life-Sciences (Lowell, MA, US). Ecoscint A scintillation solution was from National diagnostics (Hessle, England, UK). OptiPhase Supermix, UniFilter-96 GF/B, ScintiPlate-96 TC plates and all liquid scintillation was performed by 2450 MicroBeta_2_ scintillation counter, were obtained from PerkinElmer (Shelton, CT, US). Thin layer chromatography plates were purchased from Merck (Darmstadt, Germany). NuGO human Genechip arrays were obtained from Affymetrix (Santa Clara, CA, US). Seahorse XF96e analyzer, XF Base medium and XF Cell Mito Stress Test Kit were from Agilent (Wilmington, DE, US).

### Methods

#### Cell culture

Satellite cells were isolated as previously described [19] from the *m*. *obliquus internus abdominis*, *m*. *vastus lateralis* or *mm*. *interspinales* of 9 healthy donors. Donors were both male (4) and female (5), 38 ± 5 years old, had a body mass index of 22.5 ± 1.1 kg/m^2^ and fasting glucose 5.3 ± 0.3 mM. The biopsies were obtained with informed consent and approved by the National Committee for Research Ethics, Norway (S-04133 REK sør, 2011/2007 REK sør-øst B, 2015/124 REK sør-øst B). Clonetics human myoblasts isolated from two healthy female donors were used for measurement of oxygen consumtion rate (Lonza, Cologne, Germany). Skeletal muscle cells from each donor (at passage 2–4) were cultured on 12- or 96-well plates or 25 cm^2^ flasks in DMEM-Glutamax (5.5 mM glucose), 2% fetal calf serum (FCS), 2% Ultroser G, 25 IU pencillin, 25 μg/ml streptomycin, and 1.25 μg/ml amphotericin B. At 70–80% confluence, the growth medium was replaced by DMEM-Glutamax supplemented with 2% FCS, 25 IU penicillin, 25 μg/ml streptomycin, 1.25 μg/ml amphotericin B, and 25 pM insulin to induce differentiation of myoblasts to form multinucleated myotubes. Experiments were performed after 6–7 days of differentiation. The cells were cultured in humidified 5% CO_2_ atmosphere at 37°C, and the media were changed every 2–3 days. Each experiment were performed with different donors, however not all donors were used in all experiments. For the oxygen consumption rate measurements, skeletal muscle cells cultured and differentiation into myotubes were initiated by replacing the growth medium by αMEM containing 2% horse serum.

#### Scintillation proximity assay (SPA)

Radiolabeled substrates taken up and accumulated by adherent cells will be concentrated close to the scintillator embedded in the plastic bottom of each well (ScintiPlate-96 TC, PerkinElmer) and provide a stronger signal than the radiolabel dissolved in the medium alone [20]. Myotubes were cultured in 96-well ScintiPlate as described above with a mixture of 100 μM fatty acids. Measurements of fatty acids present in the cell by scintillation proximity assay (SPA) were performed in medium without phenol red with [1-^14^C]OA (0.5 μCi/ml, 9 μM) and non-labeled PA (16:0) and EPA (20:5, n-3) and were monitored for 0, 1, 2, 4, 6 and 24 h during the incubation. Thereafter, the media were changed to DPBS with 10 mM HEPES, 0.5% BSA, and 0.1 mM glucose and liquid scintillation measurements were monitored at 0, 1, 2, 4 and 6 h. The decline in [1-^14^C]OA present in the cells in the absence and presence of triacsin C (10 μM) was then studied. Triacsin C inhibits long-chain fatty acyl-CoA synthetase and will therefore inhibit, among other pathways, fatty acid re-esterification. Earlier reports in human skeletal myotubes have shown that TAG synthesis is efficiently blocked with incubation of 10 μM triacsin C (an inhibitor of fatty acid re-esterification and oxidation) for 3 h [21]. Re-esterification can be estimated as fatty acid present in the cells, calculating the difference with and without triacsin C present, as previously reported by Bezaire et al. [22]. The amount of radioactivity in the cells was related to total cell protein content measured according to Bradford [23].

#### Lipid distribution

Myotubes were treated with a mixture of 100 μM fatty acids for 24 h. The mixture was trace amounts of [1-^14^C]OA (0.5 μCi/ml, 9 μM) and non-labeled PA (16:0) and EPA (20:5, n-3). After incubation the myotubes were washed twice with PBS and harvested with two additions of 125 μl distilled water. Cellular lipids were extracted as previously described [24] by extraction of homogenized cell fraction, separation of lipids by thin layer chromatography and quantification by liquid scintillation. A non-polar solvent mixture of hexane:ether:acetic acid (65:35:1) was used to separate the lipids. The amount of neutral lipids was related to total cell protein content.

#### Acid soluble metabolites

Myotubes were treated with a mixture of 100 μM fatty acids for 24 h. The mixture was trace amounts of [1-^14^C]OA (0.5 μCi/ml, 9 μM) and non-labeled PA (16:0) and EPA (20:5, n-3). Measurement of acid soluble metabolites (ASMs) was performed using a method modified from Skrede et al. [25]. Incubation media (100 μl) were transferred to Eppendorf tube, precipitated with 300 μl HClO_4_ (1 M) and 30 μl BSA (6%), and centrifuged at 10.000 rpm (9600×*g*) for 10 min at 4°C. Then, 200 μl of the supernatant was counted by liquid scintillation. ASMs consist mainly of tricarboxylic acid cycle metabolites and reflect incomplete fatty acid oxidation, and were related to total cell protein content.

#### Substrate oxidation assay

Myotubes were cultured on 96-well CellBIND microplates. The cells were preincubated with 100 μM PA or EPA for 24 h. Then [1-^14^C]OA (0.5 μCi/ml, 100 μM) was given to the cells in DPBS (with Mg^2+^ and Ca^2+^, Gibco) with 10 mM HEPES and 1 mM L-carnitine during the 4 h CO_2_ oxidation assessment. OA was bound to BSA at a ratio of 2.5:1. A 96-well UNIFILTER microplate (PerkinElmer) was mounted on top of the CellBIND plate as previously described [20], and the cells were incubated at 37°C for 4 h. The [^14^C]CO_2_ trapped in the filter was counted by liquid scintillation, and the result reflects CO_2_ production. The remaining cell-associated radioactivity (substrate accumulated) was also assessed by liquid scintillation, and both CO_2_ and cell-associated were related to total cell protein content.

#### Measurement of oxygen consumption rate

Oxygen consumption rates (OCR) were recorded in primary human skeletal muscle cells from two different donors (Lonza) on a Seahorse XF96e analyzer. One hour before the start of the recordings, the medium was changed to Seahorse XF Base medium, supplemented with 5 mM glucose, 2 mM glutamine, 1 mM sodium pyruvate and 0.5 mM HEPES, pH 7.4. Then, OCR was recorded three times at 6 min intervals at baseline, and following injections with 5 μM oligomycin, 3 μM FCCP and 4 μM rotenone/antimycin A (XF Cell Mito Stress Test Kit), respectively. Determinant of mitochondrial function (basal respiration, proton leak, maximal respiration, spare respiratory capacity, non-mitochondrial oxygen consumption and ATP production) were calculated by the Seahorse XF Mito Stress Test Report Generator using the following formulaes: basal respiration = last rate measurement before first injection–non-mitochondrial respiration rate; proton leak = minimum rate measurement after oligomycin injection–non-mitochondrial respiration; maximal respiration = maximum rate measurement after FCCP injection–non-mitochondrial respiration); spare respiratory capacity = maximal respiration–basal respiration; non-mitochondrial oxygen consumption = minimum rate measurement after rotenon/antimycin A injection; ATP production = last rate measurement before oligomycin injection–minimum rate measurement after oligomycin injection.

#### Gene expression and bioinformatics analysis

Previously performed array was submitted to Gene Expression Omnibus (accession number: GSE18589). In short, human myotubes from three donors were cultured in 25 cm^2^ flasks and preincubated with 100 μM PA or EPA for 24 h. Thereafter the cells were harvested, RNA isolated and Affymetrix human NuGO GeneChip arrays was run [17]. Here log_2_-transformed values were imported into *Partek Genomics Suite 6*.*6*. software for analysis, corrected for donor differences and 2-way ANOVA was performed. Genes with a p<0.05 for EPA vs PA were used for GO enrichment analysis.

#### Presentation of data and statistics

All values are reported as means ± SEM. The value n usually represents the number of different donors used each with at least triplicate samples. Linear mixed models (LMM) (SPSS version 20 (IBM SPSS Statistics, Armonk, NY, US) were used to compare effects of different fatty acids over time when SPA was used in accumulation, lipolysis and re-esterification experiments. A p-value <0.05 was considered significant. Student´s t-test was also used for comparison of EPA vs PA for single data points. Data underlying the findings are presented in S1 File.

## Results

### Fatty acid accumulation was increased in myotubes during co-incubation with eicosapentaenoic acid

**Fig 1:**
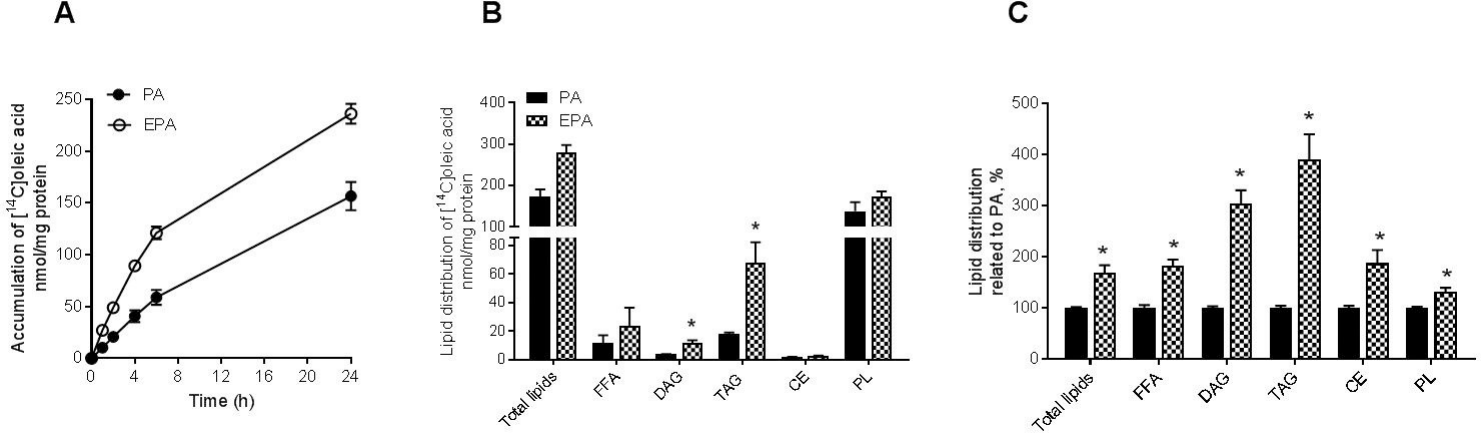
Accumulation and distribution of oleic acid in myotubes when co-incubated with fatty acids. Human myotubes were grown and differentiated in 96-well ScintiPlate or 12-well tissue culture plates. On day 6 of differentiation the myotubes were treated with a mixture of 100 μM fatty acids for 24 h. The mixture was trace amounts of [^14^C]OA (9 μM) and non-labeled PA (16:0) or EPA (20:5, n-3) (91 μM). **(A)** Cell-associated radioactivity was measured during 24 h by SPA. Real-time accumulation of radiolabel was monitored as described in Methods. Results represent mean ± SEM (nmol/mg protein) for n = 3 donors. Significant increase for EPA vs. PA (all-over effect). p<0.05 for EPA vs. PA, LMM statistical test (SPSS). **(B-C)** Lipids were separated by thin layer chromatography and quantified by liquid scintillation. Results are shown as mean ± SEM for absolute values, nmol/mg protein (B) and related to PA, % (C) from 4 individual experiments. *p<0.05 for EPA vs. PA (t-test). EPA, eicosapentaenoic acid; PA, palmitic acid; SPA, scintillation proximity assay; LMM, linear mixed model; FFA, free fatty acid; DAG, diacylglycerol; TAG, triacylglycerol; CE, cholesteryl ester; PL, phospholipids.

Myotubes were treated with mixtures of 100 μM fatty acids for 24 h and cell-associated radioactivity was measured during 24 h by scintillation proximity assay (SPA). By using non-labelled PA or EPA it was necessary to use a different fatty acid as tracer, like [^14^C]OA, to study the effect the two fatty acids had on lipid metabolism. The FA mixtures were trace amounts of [^14^C]OA (9 μM) and non-labeled PA (16:0) or EPA (20:5n-3). Co-incubation of human myotubes with eicosapentaenoic acid (EPA) markedly increased real-time accumulation of labeled OA as compared to co-incubation with PA at all time points measured (Fig 1A). The difference in accumulation between EPA and PA was established already after 1 h of co-incubation. For co-incubation experiments with [^14^C]OA and LA (18:2n-6) the effect on fatty acid accumulation was similar to the effect of PA (S1 Fig). Moreover, also when using [^14^C]PA as substrate EPA showed increased accumulation during co-incubation compared to OA and LA (S1 Fig).

### Lipid distribution was changed after co-incubation with eicosapentaenoic acid

Myotubes were treated with the two mixtures of 100 μM fatty acids as described above for 24 h and lipid distribution was measured. EPA caused a significantly higher incorporation of labeled OA into diacylglycerol (DAG) and triacylglycerol (TAG) in comparison with PA (Fig 1B). When relating the data to PA, EPA significantly increased the incorporation of labeled OA into all lipid classes (total cellular lipids, free fatty acids, phospholipids, DAG, TAG and cholesteryl ester) (Fig 1C). The greatest difference between EPA and PA was observed with a 3.9-fold increase for TAG and 3-fold for DAG, respectively. The level of unesterified oleic acid in the cells was also higher for EPA compared to PA (Fig 1C).

### Lipolysis and fatty acid re-esterification was increased after co-incubation with eicosapentaenoic acid

**Fig 2:**
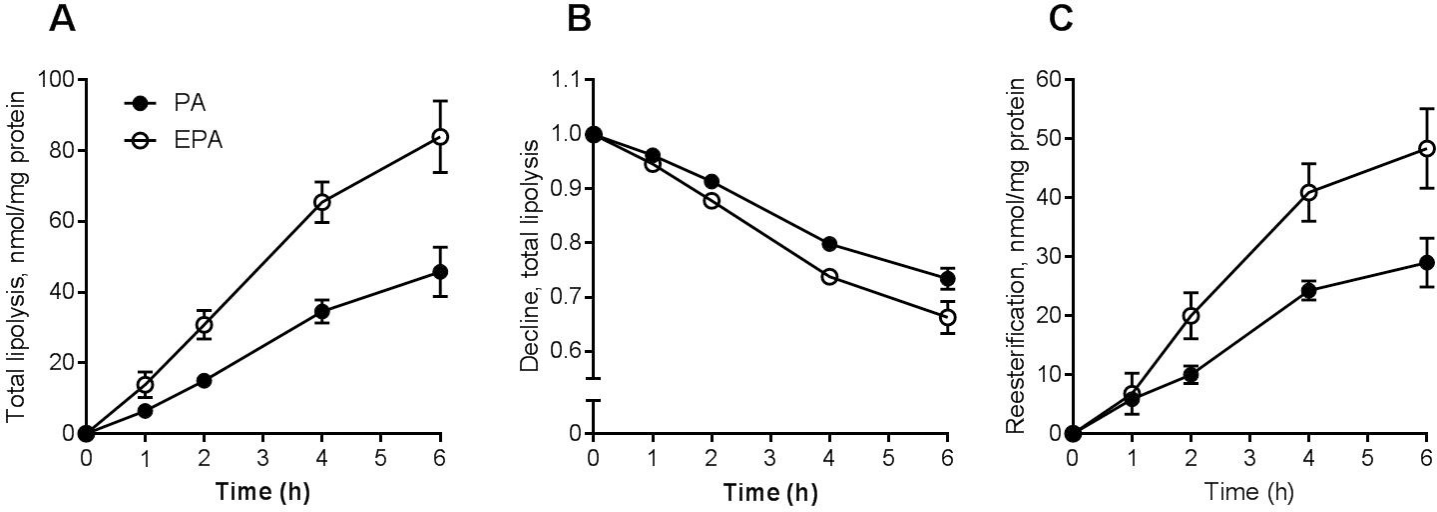
Lipolysis and re-esterification of oleic acid in myotubes after co-incubation with fatty acids. Human myotubes were grown and differentiated in 96-well ScintiPlate tissue culture plates. On day 6 of differentiation the myotubes were treated with a mixture of 100 μM fatty acids for 24 h. The mixture was trace amounts of [^14^C]OA (9 μM) and non-labeled PA or EPA (20:5, n-3) (91 μM). **(A)** Total lipolysis (lipolysis measured in presence triacsin C, 10 μM) of cell-associated [^14^C]OA at 1, 2, 4, and 6 h after 24 h pretreatment. **(B)** Total lipolysis presented as relative decline (i.e. data normalized to cell-associated radioactivity at zero time) in cell-associated [^14^C]OA at 1, 2, 4, and 6 h after 24 h pretreatment. **(C)** Re-esterification of [^14^C]OA, calculated as the difference between lipolysis measured at 1, 2, 4, and 6 h by SPA in the presence or absence of triacsin C (10 μM). Results represent mean ± SEM as nmol/mg protein **(A, C)** and relative decline in cell-associated radioactivity **(B)** for n = 3 donors. Significant increased lipolysis, decline and re-esterification for EPA vs. PA. p<0.05 for EPA vs. PA (all-over effect), LMM statistical test (SPSS). EPA, eicosapentaenoic acid; PA, palmitic acid; SPA, scintillation proximity assay; LMM, linear mixed model.

After 24 h co-incubation with PA or EPA and [^14^C]OA total lipolysis (lipolysis in presence of triacsin C) was measured by SPA at 0, 1, 2, 4 and 6 h. Total lipolysis was markedly increased by EPA compared to PA for all time points measured (Fig 2A). The relative decline in cell-associated [^14^C]OA after 24 h co-incubation was also significantly higher for EPA compared to PA indicating that lipolysis of [^14^C]OA was increased also when adjusting for increased accumulation of labeled OA in the presence of EPA (Fig 2B). After 24 h co-incubation with PA or EPA, re-esterification of [^14^C]OA was also increased by EPA during 2–6 h compared to PA (Fig 2C).

### Fatty acid beta-oxidation was increased during co-incubation with eicosapentaenoic acid

**Fig 3:**
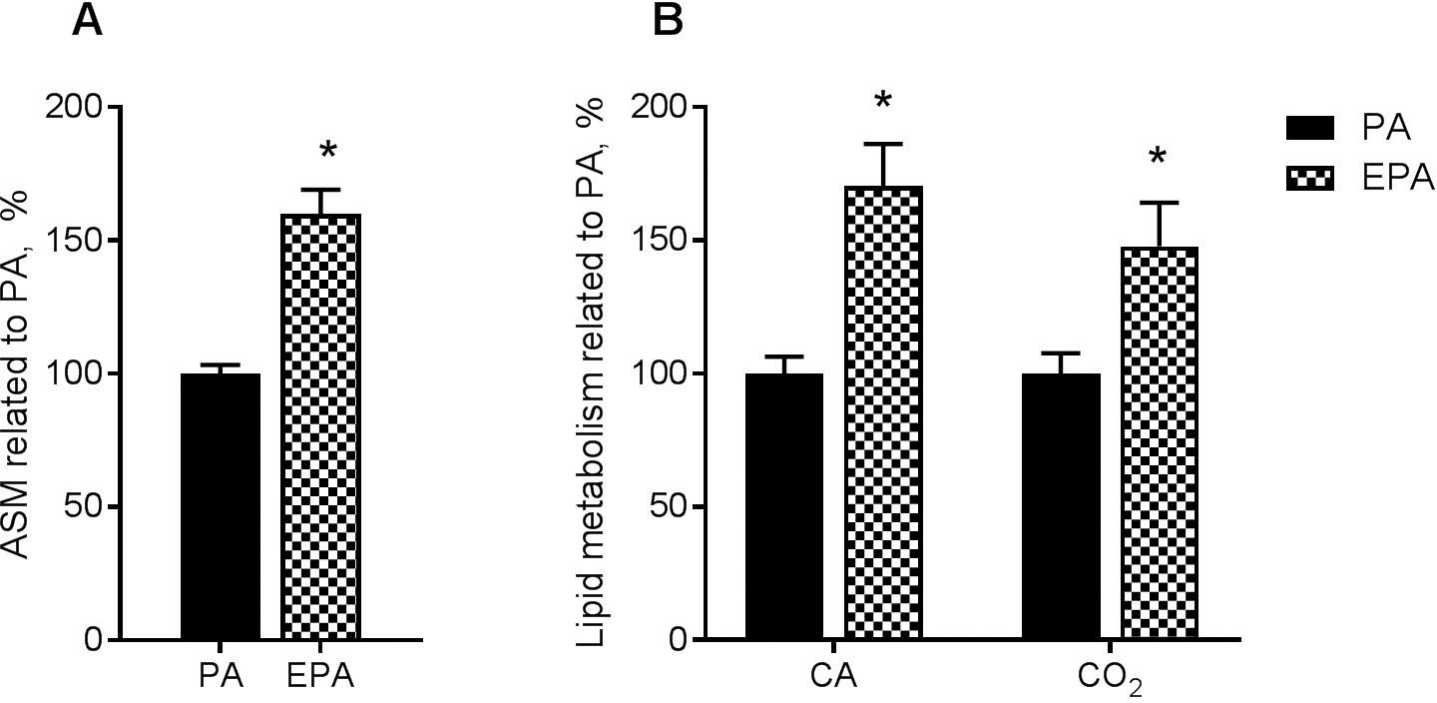
Oleic acid accumulation and oxidation after preincubation with fatty acids. **(A)** OA β-oxidation during co-incubation with fatty acids. Human myotubes were grown and differentiated in 12-well tissue culture plates. On day 6 of differentiation the myotubes were treated with a mixture of 100 μM fatty acids for 24 h. The mixture was trace amounts of [^14^C]OA (9 μM) and non-labeled PA (16:0) or EPA (20:5, n-3) (91 μM). Figure shows formation of acid-soluble metabolites (ASMs) in myotubes after 24 h. Results are shown as mean ± SEM (related to PA, %) from 4 individual experiments. Absolute values (mean ± SEM); PA: 24.5 ± 4.8; EPA: 41.3 ± 8.7 nmol/mg cell protein. **(B)** OA accumulation and complete oxidation after preincubation with fatty acids. Human myotubes were treated with fatty acids (100 μM EPA or PA) for 24 h. Thereafter, accumulation (cell-associated) and complete oxidation (CO_2_ production) from added [^14^C]OA (100 μM) was measured for 4 h. Results represent mean ± SEM, n = 3. Absolute values (mean ± SEM) for PA; CO_2_: 15.9 ± 2.3; Cell-associated: 49.9 ± 10.9 nmol/mg cell protein. *p<0.05 for EPA vs. PA (t-test). CA, cell-associated; EPA, eicosapentaenoic acid; PA, palmitic acid.

Myotubes were co-incubated with the same mixtures of fatty acids as above. Cell culture medium was collected at 24 h and fatty acid oxidation measured as acid-soluble metabolites (ASMs), which give an indication of fatty acid β-oxidation. There was a 60% increase in formation of ASM in the presence of EPA when compared to PA (Fig 3A).

### Cell-associated fatty acid and complete fatty acid oxidation were increased in myotubes after incubation with eicosapentaenoic acid

Oleic acid metabolism was also examined after exposure to 100 μM EPA or PA for 24 h (Fig 3B). Cell-associated and oxidation to carbondioxide (CO_2_) after 4 h incubation with [^14^C]OA was increased by 70% and 48% after pretreatment with EPA in comparison with PA.

### Mitochondrial respiration was increased after incubation with eicosapentaenoic acid

**Fig 4:**
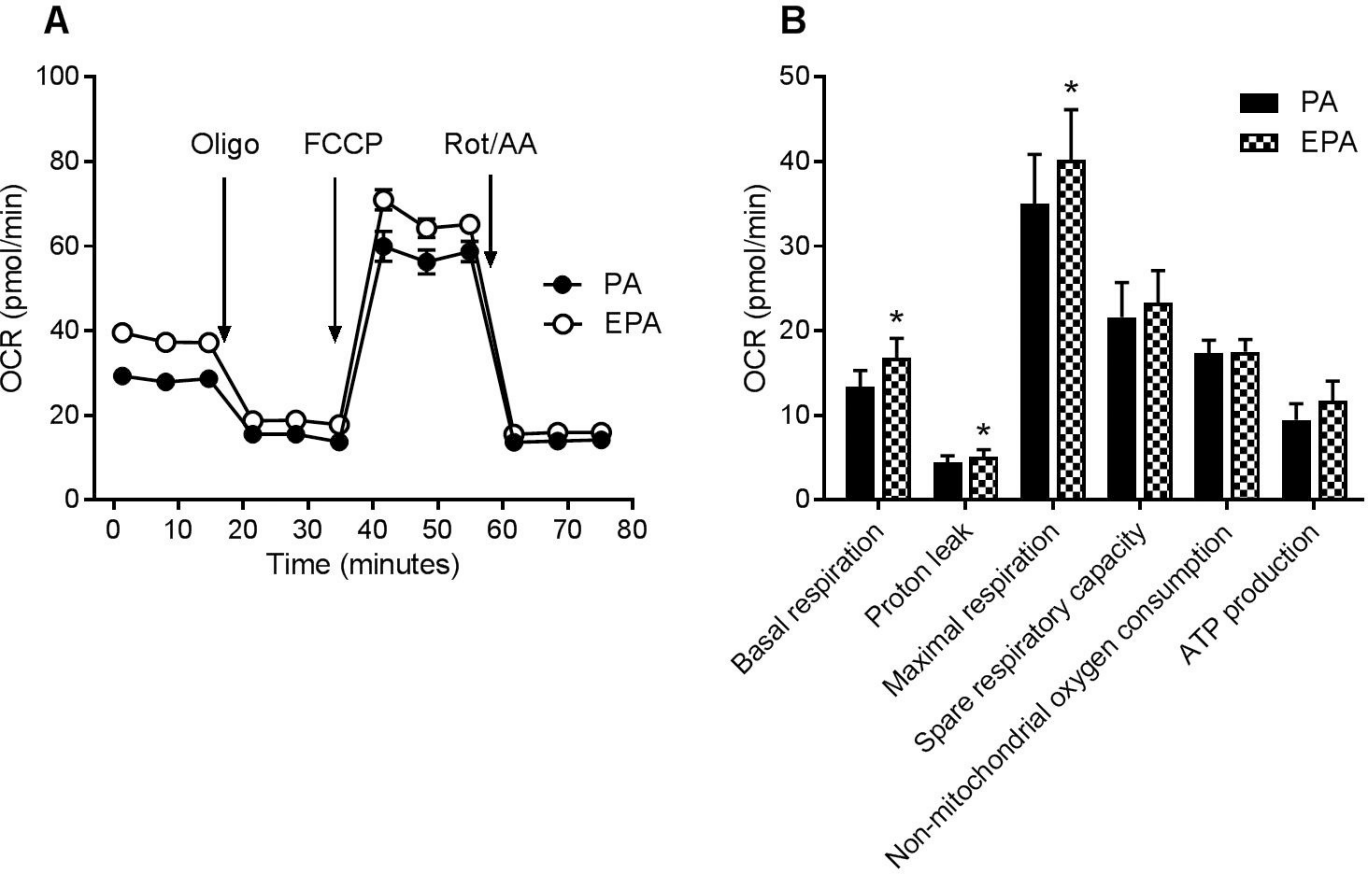
Mitochondrial function after preincubation with fatty acids. Human skeletal muscle cells were grown in 96-well Seahorse tissue culture plates, and treated with 100 μM of PA or EPA for 24 h, before measurement of oxygen consumption rate (OCR) with the Seahorse XF96e analyzer. **(A)** OCR time curve from an experiment with one of two donors. **(B)** OCR parameter calculations by the Seahorse XF Mito Stress Test Report Generator from experiments with two different donors (n = 8). Results represent mean ± SEM. *p<0.05 for EPA vs. PA (t-test). EPA, eicosapentaenoic acid; PA, palmitic acid; Oligo, oligomycin; FCCP, carbonyl cyanide-p-trifluoromethoxyphenylhydrazone; Rot, rotenone, AA, antimycin A.

Mitochondrial respiration was determined in human skeletal muscle cells using an extracellular flux analyzer (Fig 4A). Basal respiration, proton leak and maximal respiration were significantly increased (by means 30, 30 and 19%, respectively) in cells exposed for 24 h to 100 μM EPA compared to PA (Fig 4B), while ATP production tended to increase (mean increase 43%, p = 0.055).

### Microarray analysis of gene expression

**Fig 5:**
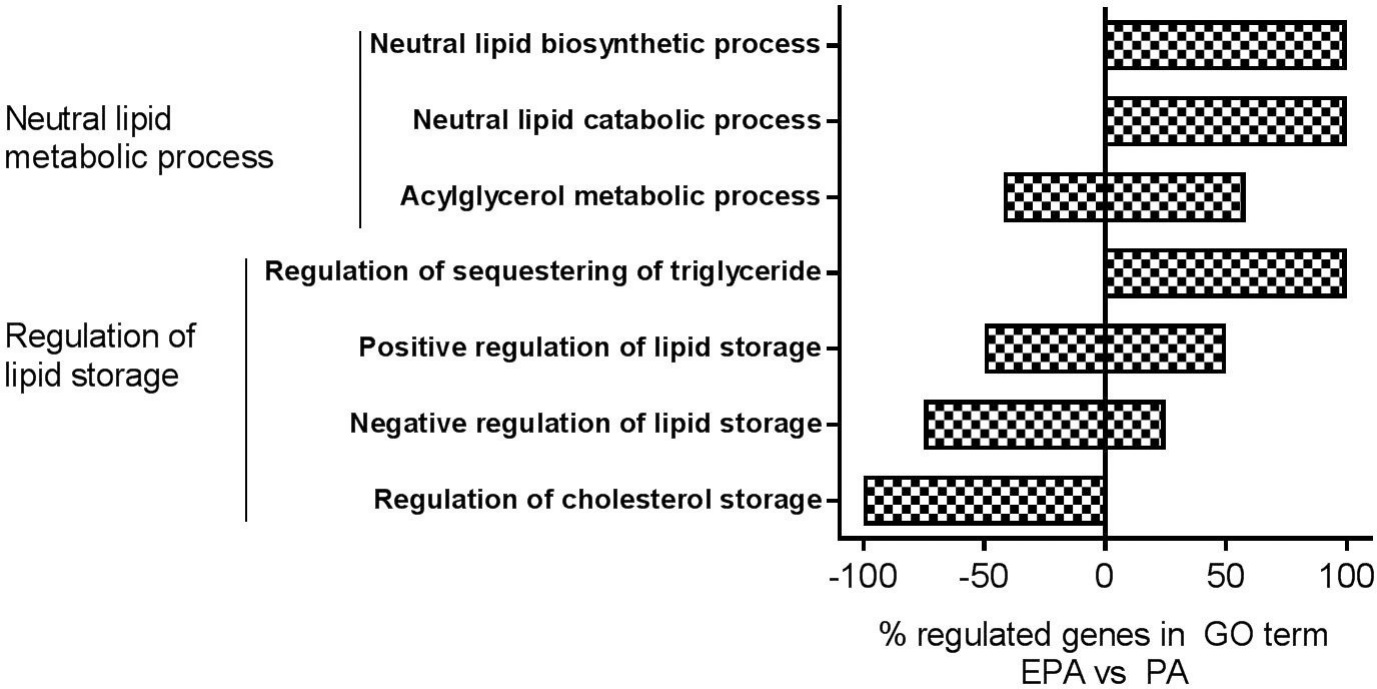
GO enrichment analysis. Myotubes were incubated with PA or EPA (100 μM) for 24 h and then harvested for RNA isolation. Affymetrix human NuGO GeneChip arrays was used to measure gene expression and analysis performed in Partek Genomics Suite 6.6 software. GO enrichment analysis revealed that the GO terms”Neutral lipid metabolic process” (GO:0006638, upper 3) and”Regulation of lipid storage” (GO:0010883, lower 4) were changed significantly (p<0.05) by EPA vs. PA. They are presented with % of genes up- or down-regulated in each child GO term and genes are presented in S1 Table. EPA, eicosapentaenoic acid; PA, palmitic acid.

Microarray analysis was performed to examine whether treatment for 24 h with 100 μM EPA regulated gene expression differently in the myotubes compared to 100 μM PA. Gene Ontology (GO) enrichment analysis revealed that, among others, the GO terms”Neutral lipid metabolic process” (GO:0006638) and”Regulation of lipid storage” (GO:0010883) were significantly changed (p<0.05) by EPA compared to PA (Fig 5). Genes involved in the GO terms in Fig 5 are listed in S1 Table. All genes that were differently regulated (EPA vs. PA) are listed in S2 Table.

### Diacylglycerol acyltransferase 1 inhibition reduced the effect of eicosapentaenoic acid to promote lipid accumulation

**Fig 6:**
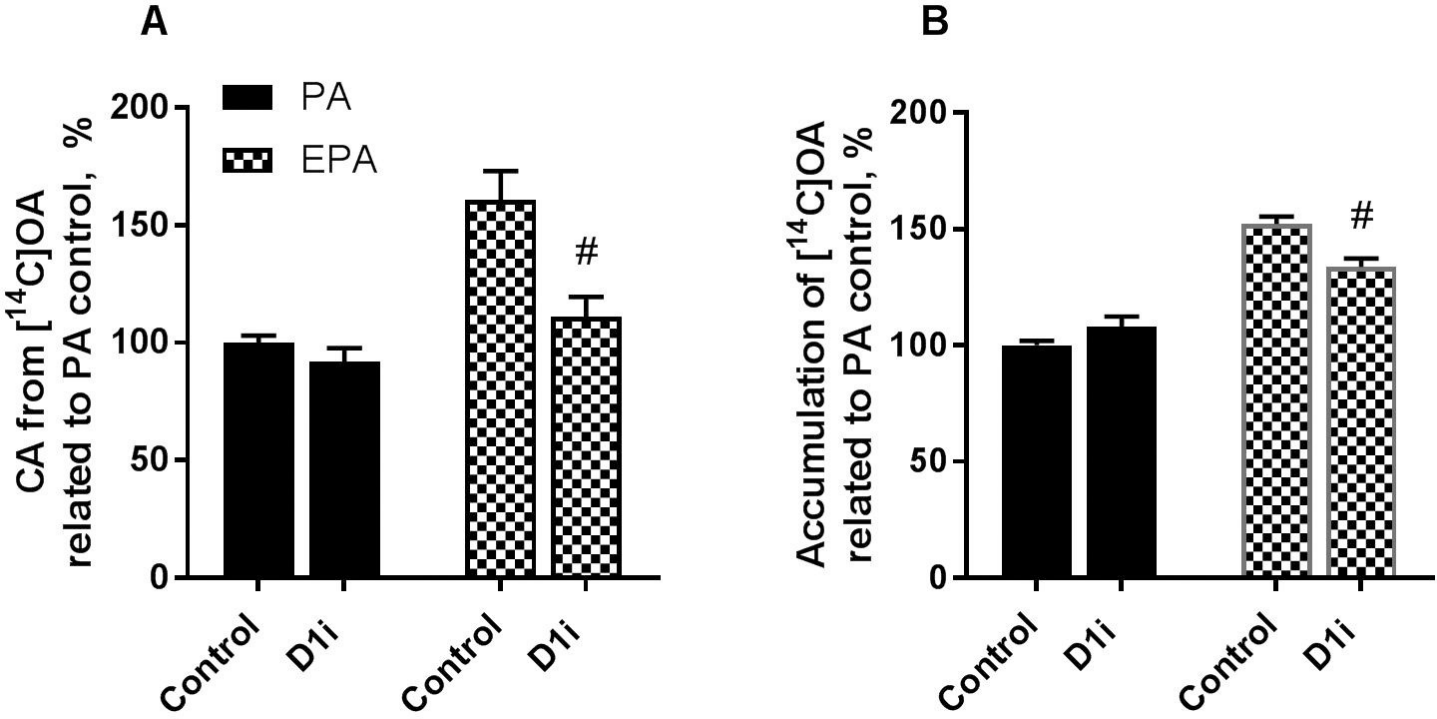
Effect of DGAT1 inhibitor on cell-associated and accumulated oleic acid. **(A)** Human myotubes were treated with fatty acids (100 μM EPA or PA) for 24 h. Cell-associated [^14^C]OA (100 μM) was measured after 4 h with and without DGAT1 inhibitor (D1i, A922500, 1 μM). Results represent mean ± SEM, from experiments with 4 different donors and 4–8 parallels (n = 26). Cell-associated PA (100%): 53.8 ± 4.0 nmol/mg cell protein. **(B)** Human myotubes were grown and differentiated in 96-well ScintiPlate tissue culture plates. On day 6 of differentiation the myotubes were treated with a mixture of 100 μM fatty acids for 24 h and cell-associated radioactivity was measured during 24 h by SPA. The mixture was trace amounts of [^14^C]OA (9 μM) and non-labeled PA or EPA (91 μM). DGAT1 inhibitor was also added for 24 h. Results represent mean ± SEM from experiments with 3 different donors and 8 parallels (n = 24). Accumulated PA (100%): 156 ± 13.8 nmol/mg cell protein. #p<0.05 for D1i inhibitor vs. control in EPA-treated cells (t-test). EPA, eicosapentaenoic acid; PA, palmitic acid; D1i, diacylglycerol acyltransferase 1 inhibitor.

To further look into mechanisms for increased TAG accumulation by EPA compared to PA we studied how the effect of a specific inhibitor of diacylglycerol acyltransferase 1 (A922500 [26]) affected lipid accumulation and turnover for 4 h (Fig 6A) or 24 h (Fig 6B). Diacylglycerol acyltransferase (DGAT) catalyse the last and dedicated step in TAG synthesis, the esterification of fatty acyl-CoA to DAG [27]. By using an inhibitor of DGAT we could determine if the effect of EPA on skeletal muscle cells was mechanistically linked to TAG accumulation and turnover. This inhibitor has previously been used in experiments with human myotubes showing 90% decrease in incorporation of [^14^C]OA into TAG [18]. DGAT1 inhibition significantly reduced cell-associated [^14^C]OA in myotubes both after preincubation with EPA (Fig 6A) and during co-incubation with EPA (Fig 6B), whereas there was no effect when cells were treated with PA.

## Discussion

In the present study our focus was to study the effects of fatty acids to modify cellular TAG—FA cycling in human skeletal muscle cells. Based on previous studies with different fatty acids we decided to examine the effect of eicosapentaenoic acid (EPA) compared to palmitic acid (PA) on real-time fatty acid accumulation, lipolysis, re-esterification and β-oxidation during co-incubation with trace amounts of labeled oleic acid (OA) in myotubes. We observed an enhanced accumulation, lipolysis and re-esterification of [^14^C]OA by co-incubation with EPA when compared to PA. Lipid distribution showed that EPA promoted increased incorporation of [^14^C]OA into various lipid classes, especially into DAG and TAG, and the level of unesterified [^14^C]OA in the cells was also higher in presence of EPA compared to PA. Furthermore, an increased fatty acid β-oxidation was seen after co-incubation with EPA. Preincubation of myotubes with EPA also increased basal and maximal mitochondrial respiration and proton leak, as well as complete oxidation of [^14^C]OA. Gene expression studies indicated that EPA, compared to PA, did alter the expression of genes involved in neutral lipid metabolism and lipid storage. Finally, the effect of EPA to promote fatty acid accumulation was reduced by inhibition of diacylglycerol acytransferase 1 (DGAT1), which catalyzes the conversion of DAG and fatty acyl-CoA to TAG.

The effect of EPA to increase uptake of fatty acids acutely (during 4 h) after pretreatment, as well as during co-incubation is in accordance with our previous observations that EPA promoted accumulation of labeled fatty acids such as OA and PA [15, 16, 18]. In the present study this effect was established already after 1 h of co-incubation. Previously, we have seen that chronic incubation of human myotubes with EPA for 24 h or 4 days, respectively, promoted uptake of both labeled OA and PA, increased TAG accumulation and fatty acid β-oxidation and reduced the levels of acyl-CoA [16]. Consistent with this, treatment with EPA increased the number of lipid droplets compared to PA in another study on human myotubes [17]. Furthermore, we have also observed that the differences between OA and PA on their cellular accumulation and lipolysis were eliminated in human myotubes when co-incubated with EPA [18].

Here we in addition observed indications of an increased TAG—FA cycling after EPA exposure in myotubes. The observation that EPA increased lipolysis and re-esterification might be a result of a higher accumulation of TAG. However, EPA also caused increased lipolysis compared to PA when adjusting for the amount of accumulated fatty acid in the cells. Moreover, fatty acid re-esterification was simultaneously increased by EPA. Combined with co-incubation data this indicates a higher enrichment in TAG as well as increased turnover of labeled fatty acids in the presence of EPA when compared to PA, but also during co-incubation with other fatty acids such as LA and OA. It was shown *in vivo* in mice that n-3 FA supplementation to a high-fat saturated diet modulated metabolic pathways of TAG synthesis, lipolysis, fatty acid oxidation and thermogenesis in skeletal muscle at mRNA and protein levels suggesting activation of TAG—FA substrate cycling [28]. To study TAG accumulation and turnover in more detail we blocked the final step in the synthesis of TAG using an inhibitor of DGAT1. Our data showed that DGAT1 inhibition counteracted EPA´s effect to promote OA accumulation, indicating that the effect of EPA on fatty acid metabolism and lipid turnover may be dependent of TAG synthesis and possibly be mediated through increased TAG—FA cycling. Accordingly, there was no effect of DGAT1 inhibition on OA accumulation after co-incubation with PA. In another study, increased fatty acid uptake was shown to increase futile cycling of fatty acids into TAG in C2C12 mice skeletal muscle cells [13]. They observed that cycling of fatty acids was important for maintaining a low TAG content and insulin responsiveness of the cells [13]. Futile cycling of TAG has been described in adipocytes by induction of glycerol kinase and glycerogenesis mediated by PGC-1 alpha and PPARs activation [11, 12, 29]. It is known that n-3 fatty acids activates PPAR isoforms [30–32], although their affinity for the receptors may vary. However, we have previously observed that various fatty acids (PA, OA, LA and EPA) increase mRNA expressions of PPAR-regulated genes in skeletal muscle cells compared to a fatty acid-free control [17]. This is likely because both saturated and unsaturated fatty acids are ligands for PPAR activation [33], which further might explain why there was only small effects on PPAR-regulated genes in our microarray gene expression analysis when EPA was compared with PA.

In the present study mitochondrial respiration and proton leak was increased in myotubes after exposure to EPA for 24 h. We have previously observed that pretreatment of human myotubes with EPA promoted fatty acid β-oxidation, while also stimulating glucose transport and oxidation compared to OA [15, 16]. There are also *in vivo* studies that suggest increased skeletal muscle mitochondrial function after n-3 FA supplementation [28, 34]. Ten weeks of EPA supplementation in aging mice improved mitochondrial oxidative capacity and bioenergetic efficiency in skeletal muscle, however supplementation of another n-3 FA, docosahexaenoic acid (DHA), had no effect [34]. In a study in older human adults, 16 weeks of n-3 PUFA supplementation did not change skeletal muscle mitochondrial respiration, but reduced mitochondrial ROS production [35], neither did supplementation of EPA plus DHA for 6 months change muscle mitochondrial function in insulin-resistent non-diabetic humans [36]. However, in young, healthy adults 12 weeks of EPA plus DHA supplementation improved mitochondrial membrane composition and ADP kinetics in skeletal muscle [37].

In our study, we compared expression of genes induced by EPA related to PA, and the fold changes found (S2 table) are in general small (e.g. carnitine palmitoyltransferase 1A (CPT1A), perilipin 2 (PLIN2) and monoglyceride lipase (MGLL) with fold change 1.2–1.4) or opposite of expected (e.g. cluster of differentiation 36/fatty acid translocase (CD36) fold change -1.33). Therefore, GO enrichment analysis from the microarray mRNA expression analysis was exerted to get a more overall picture of the biological processes that may be modified differently by the two fatty acids. GO enrichment analysis revealed that EPA compared to PA enhanced only some biological processes involved in neutral lipid biosynthesis and catabolic processes. Among GO terms under “Regulation of lipid storage”, “Regulation of sequestering of triglyceride” was increased and “Negative regulation of lipid storage” was decreased by EPA. Among GO terms under “Neutral lipid metabolic process”, both “Neutral lipid biosynthetic process” and “Neutral lipid catabolic process” were increased by EPA. This, as well as the acute effect of EPA to promote fatty acid accumulation during co-incubation, suggest that the effects of EPA on lipid turnover and oxidation in part may be mediated by changes in gene expression and that non-genomic mechanisms also may be involved [38, 39]

Skeletal muscle is the largest organ and a major contributor to basal metabolic rate. Increasing energy expenditure in muscle through non-shivering thermogenic mechanisms such as TAG—FA cycling could substantially affect whole body metabolism and body weight gain [40, 41]. Although heat production from muscle has long been recognized as a thermogenic mechanism, whether muscle can produce heat independently of contraction remains controversial. Thus, current evidence does not indicate a clear role of skeletal muscle in non-shivering thermogenesis, which may be due to lack of methods allowing measurement of these processes separately from other muscle thermogenic processes [40]. We believe our cell model is a valuble tool to study some of these procesess in more detail.

In conclusion, findings from this study suggest an increased TAG—FA turnover i.e. futile substrate cycling in human myotubes induced by EPA, combined with increased fatty acid oxidation and mitochondrial function. Our data indicate that enhancing these processes could be of importance for the potential favourable effects of long-chain n-3 fatty acids on skeletal muscle as well as whole-body energy metabolism.

## Supporting information

S1 File Data underlying the findings. Absolute data from each experiment.
(XLSX)

S1 Fig Real time fatty acid accumulation in myotubes when co-incubated with various fatty acids. Human myotubes were grown and differentiated in 96-well ScintiPlate. On day 6 of differentiation the myotubes were treated with a mixture of 100 μM fatty acids for 24 h and cell-associated (CA) radioactivity was measured during 24 h by SPA. The mixture was trace amounts of [^14^C]OA (9 μM) and non-labeled PA (16:0) and LA (18:2, n-6) **(A)**, or or trace amounts of [^14^C]PA (9 μM) and non-labeled OA (18:1, n-9), LA (18:2, n-6) and EPA (20:5, n-3) **(B)**. Results represent mean ± SEM for n = 5–7 donors related to PA or OA at 24 h in percent (70 ± 17 nmol/mg for PA **(A)** and 107 ± 54 nmol/mg for OA **(B))**. Significant increase for EPA vs. OA/LA (all-over effect). p<0.05 for EPA vs. OA/LA, LMM statistical test (SPSS). EPA, eicosapentaenoic acid; LA, linoleic acid; OA, oleic acid; PA, palmitic acid; SPA, scintillation proximity assay; LMM, linear mixed model.
(TIF)

S1 Table Genes changed by EPA vs. PA in myotubes. Genes in the GO terms”Neutral lipid metabolic process” (GO:0006638) and”Regulation of lipid storage” (GO:0010883) that is changed (p<0.05) for EPA vs. PA are presented. Myotubes from three donors were incubated with PA or EPA (100 μM) for 24 h and then harvested for RNA isolation. Gene expression was measured by Affymetrix human NuGO GeneChip arrays and analysis performed in *Partek Genomics Suite 6*.*6*. software. EPA, eicosapentaenoic acid; PA, palmitic acid.
(DOCX)

S2 Table Genes changed by EPA vs. PA in myotubes. Genes that is changed (p<0.05) for EPA vs. PA are presented. Myotubes from three donors were incubated with PA or EPA (100 μM) for 24 h and then harvested for RNA isolation. Gene expression was measured by Affymetrix human NuGO GeneChip arrays and analysis performed in *Partek Genomics Suite 6*.*6*. software. EPA, eicosapentaenoic acid; PA, palmitic acid.
(XLSX)

## Abbreviations

ASMs: acid soluble metabolites
BSA: bovine serum albumin
DGAT: diacylglycerol acyltransferase
DHA: docosahexaenoic acid
EPA: eicosapentaenoic acid
FA: fatty acid
GO: Gene Ontology
LA: linoleic acid
OA: oleic acid
OCR: oxygen consumption rate
PA: palmitic acid
PPAR: peroxisome proliferator-activated receptor
SPA: scintiallation proximity assay
