# Supplementary figures and images for: Increased triacylglycerol - Fatty acid substrate cycling in human skeletal muscle cells exposed to eicosapentaenoic acid
Source: PLoS One. 2018 Nov 29;13(11):e0208048. doi: 10.1371/journal.pone.0208048 (PMC6264501; doi:10.1371/journal.pone.0208048)

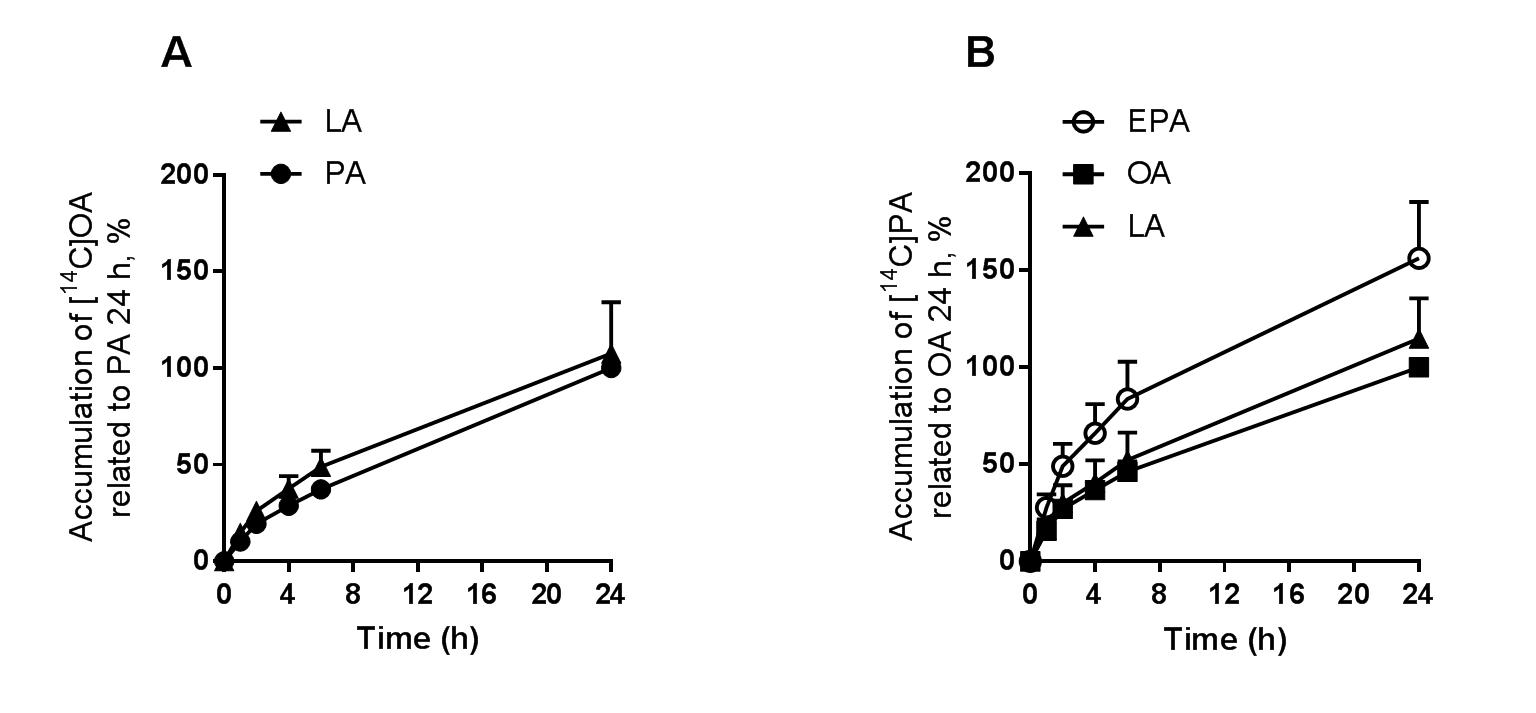

Supplement: S1 Fig — Human myotubes were grown and differentiated in 96-well ScintiPlate. On day 6 of differentiation the myotubes were treated with a mixture of 100 μM fatty acids for 24 h and cell-associated (CA) radioactivity was measured during 24 h by SPA. The mixture was trace amounts of [14C]OA (9 μM) and non-labeled PA (16:0) and LA (18:2, n-6) (A), or or trace amounts of [14C]PA (9 μM) and non-labeled OA (18:1, n-9), LA (18:2, n-6) and EPA (20:5, n-3) (B). Results represent mean ± SEM for n = 5–7 donors related to PA or OA at 24 h in percent (70 ± 17 nmol/mg for PA (A) and 107 ± 54 nmol/mg for OA (B)). Significant increase for EPA vs. OA/LA (all-over effect). p<0.05 for EPA vs. OA/LA, LMM statistical test (SPSS). EPA, eicosapentaenoic acid; LA, linoleic acid; OA, oleic acid; PA, palmitic acid; SPA, scintillation proximity assay; LMM, linear mixed model. (TIF) [file pone.0208048.s002.tif]
