# Supplementary material for: Increased triacylglycerol - Fatty acid substrate cycling in human skeletal muscle cells exposed to eicosapentaenoic acid
Source: PLoS One. 2018 Nov 29;13(11):e0208048. doi: 10.1371/journal.pone.0208048 (PMC6264501; doi:10.1371/journal.pone.0208048)
Supplement: S1 Table — Genes in the GO terms”Neutral lipid metabolic process” (GO:0006638) and”Regulation of lipid storage” (GO:0010883) that is changed (p<0.05) for EPA vs. PA are presented. Myotubes from three donors were incubated with PA or EPA (100 μM) for 24 h and then harvested for RNA isolation. Gene expression was measured by Affymetrix human NuGO GeneChip arrays and analysis performed in Partek Genomics Suite 6.6. software. EPA, eicosapentaenoic acid; PA, palmitic acid. (DOCX) [file pone.0208048.s003.docx]

| **Gene name** | **p-value** | **Fold-Change**  **(EPA vs PA)** | **Description** |
| --- | --- | --- | --- |
| LPCAT1 | 0,001 | 1,63 | Lysophosphatidylcholine acyltransferase |
| IL6 | 0,008 | 1,46 | Interleukin 6 |
| APOBR | 0,006 | 1,32 | Apolipoprotein B receptor |
| CPT1A | 0,035 | 1,24 | Carnitine palmitoyltransferase 1A |
| ABHD2 | 0,001 | 1,23 | Abhydrolase domain containing 2 |
| MGLL | 0,004 | 1,22 | Monoglyceride lipase |
| LIPF | 0,038 | 1,18 | Lipase F, fastric type |
| CIDEA | 0,043 | 1,18 | Cell death activator CIDE-A |
| AGPAT2 | 0,017 | 1,10 | 1-acyl-sn-glycerol-3-phosphate acyltransferase beta |
| ACSL1 | 0,027 | -1,17 | Long-chain-fatty-acid-CoA ligase 1 |
| NKX2-3 | 0,025 | -1,20 | Homeobox protein Nkx-2.3 |
| CD36 | 0,011 | -1,33 | Cluster of differentiation 36/fatty acid translocase |
| NR1H3 | 0,037 | -1,51 | Nuclear receptor subfamily 1 group H member 3/liver X receptor alpha |
| PCSK9 | 0,003 | -1,54 | Proprotein convertase subtilisin/kexin type 9 |
| CAV3 | 0,000 | -1,54 | Caveolin-3 |
| MEST | 0,005 | -1,59 | Mesoderm-specific transcript homolog protein |
| FBXW7 | 0,000 | -1,62 | F-box/WD repeat-containing protein 7 |
| MTTP | 0,018 | -1,63 | Microsomal triglyceride transfer protein large subunit |
| ABCA1 | 0,000 | -2,95 | ATP-binding cassette transporter |

**Supporting information table 1. Genes changed by EPA vs PA in myotubes.** Genes in the GO terms ”Neutral lipid metabolic process” (GO:0006638) and ”Regulation of lipid storage” (GO:0010883) that is changed (p<0.05) for EPA vs PA are presented. Myotubes from three donors were incubated with PA or EPA (100 μM) for 24 h and then harvested for RNA isolation. Gene expression was measured by Affymetrix human NuGO GeneChip arrays and analysis performed in Partek*^®^* Genomics Suite*^®^ 6.6.* software. EPA, eicosapentaenoic acid; PA, palmitic acid.
